# Perspective: from pathogenesis to treatment in veterinary science through organoid disease modelling

**DOI:** 10.3389/fvets.2026.1807098

**Published:** 2026-04-08

**Authors:** Bo Chen, Ron Slocombe, Smitha Rose Georgy

**Affiliations:** Section of Anatomic Pathology, Faculty of Science, Melbourne Veterinary School, University of Melbourne, Werribee, VIC, Australia

**Keywords:** checkpoint, context of use, mechanistic investigation, organoid, target discovery, veterinary disease modelling

## Abstract

Organoid technology is emerging as a powerful platform for *in vitro* disease modelling in veterinary research. Although veterinary organoid models have been established across multiple species and organ systems, most studies remain largely descriptive, with a predominant focus on model development rather than disease mechanisms or therapeutic relevance. This Perspective proposes a structured framework to guide the application of organoid models from investigations of disease pathogenesis through to therapeutic discovery. Using a canine epidermal organoid model of atopic dermatitis as an illustrative example, key principles for model complexity selection, pathology-informed characterization, and the integration of functional testing with omics-based discovery are outlined. This framework aims to highlight the mechanistic depth and translational impact of veterinary organoid research.

## 1 Introduction: veterinary organoid technology

Organoids are miniaturized, *in vitro* organ-like structures that faithfully recapitulate many characteristics of their *in vivo* organ of origin (1). They possess two essential features: (1) their derivation from stem cells, and (2) their intrinsic ability to self-organize into organotypic three-dimensional (3D) structures, distinguishing them from other *in vitro* systems such as spheroids (lack stem cell-driven self-renewal) and air-liquid interface (ALI) cultures (require manual assembly rather than spontaneous self-organization) (2). It is widely recognized that organoid systems represent a major technological advance, providing a biologically relevant bridge between conventional two-dimensional (2D) cell cultures and *in vivo* animal models, while helping to reduce animal use in accordance with the 3Rs (Replacement, Reduction, and Refinement) principles (2, 3).

Over the past decade, veterinary organoid research has expanded across a wide range of species and organ systems, including companion animals, livestock, and avian models (4–6). This expansion has been driven largely by methodological advances adapted from human biomedical research, combined with growing interest in comparative and One Health approaches (7). Canine organoids, particularly tumoroids, represent the current state-of-the-art in translational potential within veterinary medicine, driven by the clinical need for comparative oncology models and personalized therapeutic strategies (8). However, many veterinary organoid studies remain primarily focused on structural fidelity, lineage marker expression, and baseline functional properties. While these efforts provide essential foundational knowledge, they offer limited insight into disease-relevant mechanisms or therapeutic applications (Table 1). This imbalance reflects a broader potential in veterinary research, where technological adoption may outpace the development of disease-oriented experimental frameworks. In particular, early-stage veterinary researchers often encounter organoid technology as a set of experimental tools rather than as components of an integrated strategy for addressing biological questions. Without clear guidance on model selection, validation, and interpretation, organoid-based studies risk becoming descriptive endpoints rather than platforms for mechanistic and therapeutic discovery.

**Table 1:** Veterinary organoid disease models classified by disease category.

Disease type		No.	Modelled disease	Species	Organ origin	Ref.	Descriptive	Mechanistic	Therapeutic
Infectious disease	Viral	1	Caprine arthritis-encephalitis	Goat	Mammary gland	(47)	✔		
		2	Porcine respiratory coronavirus infection	Pig	Trachea	(16)	✔	○	
		3	Swine influenza	Pig	Airway (bronchial epithelial cells)	(18)	✔		
		4	Porcine epidemic diarrhea (PEDV)	Pig	Intestine (duodenum, jejunum, ileum and colon)	(27, 30, 39, 48, 49)	✔	✔	✔
		5	Porcine deltacoronavirus (PDCoV) infection	Pig	Intestine (anterior duodenum, jejunum, and ileum)	(50)	✔		
		6	Transmissible gastroenteritis (TGEV infection)	Pig	Intestine (jejunum, ileum)	(26–28, 31, 36, 51)	✔	✔	✔
		7	Mammalian orthoreovirus type 3 (MRV3) infection	Pig	Intestine (jejunum)	(52)	✔		
		8	Feline infectious peritonitis (FIP)	Cat	Intestine (ileum and colon)	(53)	✔		
		9	Bovine Rotavirus (Group A Rotaviruses) infection	Ox	Intestine (ileum)	(34)	✔	✔	
		10	Bovine herpesvirus-1 (BHV-1) infection	Ox	Trachea	(54)	✔		
		11	Rabbit haemorrhagic disease	Rabbit	Intestine (duodenum, jejunum, and ileum), hepatobiliary tissue	(55, 56)	✔		
		12	Avian influenza	Chicken	Small intestine, trachea	(57, 58)	✔		
	Bacterial	13	Proliferative enteropathy (*Lawsonia intracellularis* infection)	Pig	Intestine (ileum)	(59)	✔		
		14	Salmonellosis	Pig, Ox, Chicken	Intestine (proximal jejunum, cecum, ileum)	(60, 61)	✔		
		15	*Escherichia coli* infection	Pig, Dog, Ox	Intestine (duodenum, jejunum, ileum, rectum and colon)	(21, 37, 38, 62, 63)	✔	○	
		16	*Streptococcus suis* infection	Pig	Ileum	(64)	✔		
		17	*Mycoplasma bovis* infection	Ox	Trachea	(65)	✔		
		18	*Enterococcus cecorum* infection	Chicken	Small intestine and caeca	(66)	✔		
	Host-microbiota interaction	19	Swine nasal microbiota (*Rothia nassimurium UK1-9, Moraxella pluranimalium LG6-2* and *non-virulent Glaesserella parasuis F9*)	Pig	Nasal Turbinates	(67)	✔		
	Parasitic	20	Toxoplasmosis (*Toxoplasma gondii* infection)	Pig, Ox	Intestine (proximal jejunum)	(60)	✔		
		21	Cryptosporidiosis (*Cryptosporidium parvum* infection)	Ox	Intestine (ileum)	(68)	✔		
		22	Coccidiosis (*Eimeria tenella* infection)	Chicken	Small intestine	(69)	✔		
		23	Nematodes (*Parascaris univalens, cyathostominae* and *Strongylus vulgaris*)	Horse	Small intestine	(70)	✔		
Inflammatory/immune-related		24	Inflammatory bowel disease	Dog, Sheep	Intestine (small intestine and colon)	(22, 40, 71–74)	✔	○	
		25	Atopic dermatitis	Dog	Skin	(9, 24)	✔	○	
Cancer (patient-derived organoid, PDO)		26	Mast cell tumor	Dog	Cancer	(22)	✔		
		27	Prostate cancer	Dog	Cancer	(75)	✔		
		28	Bladder cancer	Dog	Cancer	(29, 76–78)	✔	✔	✔
		29	Bladder cancer, mammary and skin tumors, lung cancer, and melanoma	Dog, Cat	Cancer	(79)	✔		
		30	Follicular cell thyroid carcinoma	Dog	Cancer	(80)	✔		
		31	Medullary thyroid carcinoma	Dog	Cancer	(81)	✔		
		32	Lung adenocarcinoma	Dog	Cancer	(82)	✔		
		33	Mammary tumor	Dog, Cat	Mammary tumor and non-neoplastic mammary tissue	(32, 33, 83, 84)	✔	✔	✔
		34	Apocrine gland anal sac adenocarcinoma	Dog	Cancer	(85)	✔		
		35	Pheochromocytoma	Dog	Normal adrenal medulla and tumor	(86)	✔		
		36	Intestinal adenocarcinoma	Cat	Cancer	(71, 87)	✔		
		37	Nasal adenocarcinoma	Sheep	Cancer	(88)	✔		
Metabolic diseases		38	Copper storage disease	Dog	Liver	(45, 89)	✔		✔
		39	Hepatic steatosis	Cat	Liver	(90, 91)	✔		✔
Toxicity		40	Deoxynivalenol-induced toxicity	Sheep	Intestine (jejunum)	(23)	✔	○	

No., number; Ref., reference(s); ○, mechanism-association without direct causal validation.

In this Perspective, we propose a structured framework for progressing from organoid-based disease modelling toward mechanistic understanding and therapeutic exploration in veterinary science. By integrating insights from recent veterinary organoid literature with practical experience gained from developing and applying a canine epidermal organoid model of atopic dermatitis, we outline key conceptual principles that guide model establishment, validation, target discovery, and translational interpretation. This framework is intended not to prescribe specific technologies, but to facilitate question-driven research design and to lower the conceptual barrier for early-stage veterinary scientists seeking to use organoid systems for meaningful disease investigation.

## 2 From simple to complex: concepts guiding use of organoid models

Many veterinary organoid systems are currently designed as simplified models composed of a single dominant cell lineage. For example, canine epidermal organoids are typically established as keratinocyte-only cultures (9). When compared with full-thickness skin constructs or air-liquid interface (ALI) models that incorporate dermal or immune components, such systems may appear to represent a step backward in terms of architectural complexity. In veterinary organoid research, increased model complexity is often implicitly equated with greater biological relevance; however, this assumption is not always justified. Conversely, reduced complexity can represent a deliberate and strategic design choice. Simplified organoid models provide a controlled experimental setting in which epithelial-intrinsic responses to defined stimuli or treatments can be directly interrogated. This reductionist approach enhances interpretability, limits confounding variables, and facilitates clearer attribution of observed effects to specific biological pathways. Accordingly, introducing additional layers of complexity through co-culture systems, advanced differentiation protocols, or engineered microenvironments may obscure causal relationships and increase experimental cost, technical burden, and batch-to-batch variability if not guided by a clear biological question.

Different research questions, therefore, require different levels of model complexity. For studies focused on epithelial-intrinsic disease mechanisms, simplified organoid systems may be sufficient or even preferable. In other contexts, such as immune-organ interactions or questions involving microfluidics and physiology, more sophisticated model systems may be required (10, 11). Progression from simple to complex should thus be viewed not as a linear hierarchy, but as a series of informed choices driven by specific experimental objectives. Within this framework, the concept of ‘minimum viable complexity’ offers a practical and cost-effective guide for organoid-based disease modelling. Selecting the simplest model capable of addressing the biological question of interest helps preserve interpretability and reproducibility, while avoiding unnecessary technical complexity. In this sense, taking a step backward in model design may ultimately enable more meaningful steps forward in mechanistic understanding.

## 3 Model establishment and characterization: pathology as a checkpoint

One of the principal strengths of organoid-based disease modelling is its ability to preserve disease-relevant features within a three-dimensional *in vitro* context (1). This capability closely aligns with the interpretative expertise of pathologists, whose work is fundamentally grounded on evaluating structure–function relationships during disease progression (12). In veterinary science, pathological observation remains a cornerstone of research, diagnosis, and model validation. Although molecular and omics-based technologies are increasingly accessible including a recent transcriptomic study using bulk RNA sequencing and single-cell RNA sequencing of multiple canine organoids (13), their routine application is often constrained by cost. Under these conditions, morphology-based pathological assessment provides a unifying and cost-effective framework. Accordingly, pathology-informed workflows are central to the establishment and characterization of *in vitro* organoid disease models that aim to faithfully and reproducibly represent key disease features in veterinary research.

At the earliest stage of organoid generation, the appropriate selection of starting material is a critical determinant of organoid establishment and characterization. Animal tissues are often heterogeneous, particularly patient-derived cancer organoids, hence clear identification of stem cells is necessary from the outset. Molecular markers play a critical role in characterizing cell identity. Routine collection of site-matched tissue for haematoxylin and eosin (H&E) examination by pathologists prior to organoid culture provides a morphological reference for interpreting organoid architecture and differentiation, thereby supporting consistent characterization across samples.

Beyond initial establishment, a central challenge in organoid-based modelling is determining whether observed changes reflect true disease-related effects or are caused by technical factors. Culture conditions, including extracellular matrix composition, culture systems (single or multiple cell lineages), and growth factor formulations, actively shape organoid morphology, differentiation, and molecular profiles. Even after protocol optimization and standardization, batch-to-batch variations may occur and interact with inter-individual biological heterogeneity. For example, during the development of canine epidermal organoids, final organoid density and size varied across cultures, in part due to differences in stem cell viability associated with donor animals and anatomical sampling sites, as well as unavoidable variability in tissue collection. In this context, pathological evaluation is essential for comparing organoids across batches and conditions and for distinguishing biologically meaningful disease-related changes from culture-caused variability.

At the level of phenotype definition and interpretation, pathological assessment provides a structured framework for interpreting organoid phenotypes within a tissue-relevant biological context. Conventional histological evaluation, complemented by appropriate special stains (e.g., Periodic acid-Schiff, Alcian blue, Masson’s trichrome), provides essential information on tissue architecture, cellular polarity, stratification, and differentiation status. These assessments can be further strengthened by immunohistochemistry and immunofluorescence analyses to define lineage commitment, spatial organization, and pathway activation at the protein level. It is worth noting in the immunostaining that phospho-epitopes are prone to alteration during specimen handling and fixation (14). Consequently, for reliable assessment of protein kinase cascade phosphorylation, western blotting plays a critical role in minimizing false-negative results. Confocal microscopy is particularly well suited for organoid systems, as it enables high-resolution, three-dimensional visualization while preserving spatial context for molecular interpretation. Ultrastructural examination by transmission electron microscopy allows detailed evaluation of subcellular features, such as cell junctions, basement membrane formation, and organelle integrity. Scanning electron microscopy provides detailed information on organoid surface architecture and is particularly useful for visualizing organoid cocultures with microbes (15). Accurate interpretation also requires an appreciation of differentiation dynamics, as stem and progenitor cells may undergo variable lineage commitment under different culture conditions. Consequently, marker-based validation of differentiation rather than assumption based solely on tissue origin is necessary. A classic example is provided by airway organoids, which may be derived from tracheal, bronchial, or pulmonary tissues (16–18). Appropriate validation of epithelial differentiation is required to accurately define organoid identity, and more precise organoid classification is warranted rather than the generic designation of ‘airway organoid’.

Finally, in high-throughput experimental settings, morphological evaluation plays a foundational role in quality control. Evaluation of structural integrity, cellular polarity, and differentiation consistency across batches supports model standardization and reproducibility. The development and integration of advanced tools, such as high-throughput fluorescence imaging (19), further enable precise disease modelling by facilitating sensitive, high-resolution, and scalable monitoring of organoid differentiation and disease-related phenotypes over time.

Importantly, complete replication of *in vivo* pathology is neither expected nor required *in vitro.* Rather, pathologists help define which disease-relevant features are appropriately captured and can be meaningfully interrogated using organoid models, based on integrated assessment of morphology and biological context. Integration of these approaches provides a complementary and efficient strategy for organoid model characterization.

## 4 Linking mechanistic investigation to target discovery: translational considerations

Once an organoid disease model has been appropriately characterized at the morphological level, it can be used to support disease-relevant mechanistic investigation and target discovery. At this stage, the central research question shifts from whether the model is biologically meaningful to how it can be used to generate mechanistic and translational insights.

Omics-based approaches, such as transcriptomics, proteomics, genomics and metabolomics, are commonly used as initial discovery tools (20). Omics approaches are well suited for identifying dysregulated pathways and generating candidate targets. Among these, RNA sequencing is the most widely applied in veterinary research due to its accessibility and established analysis pipelines. But transcript levels often show limited concordance with protein expression, and protein-level readouts are generally regarded as being closer to the disease mechanisms (20). Moreover, across the published veterinary research (16, 21–24), most omics-based data remain correlative, or mechanism-associated descriptions rather than causal relations. In our study (24), Th2 cytokine-treated and untreated canine epidermal organoids were generated to define Th2-driven transcriptomic signatures and to identify candidate atopy-associated targets using RNA sequencing, with the aim of informing subsequent mechanistic investigations and targeted therapeutic development. Importantly, disease mechanisms are not merely statistical associations between molecular changes and phenotypes, but instead reflect causal biological processes that drive disease development (25). However, most veterinary organoid studies to date remain descriptive or hypothesis-generating, with relatively few addressing disease mechanisms in a causal and deeply mechanistic manner (Table 1). Future studies should place greater emphasis on mechanistic approaches in veterinary organoid systems.

Accordingly, functional testing represents an indispensable step in linking candidate molecular targets to disease mechanism. In organoid systems, this may include exogenous modulation (26–29), pathway- or ligand-based stimulation (30, 31), or genetic manipulation (32, 33). For instance, in feline and human breast cancer organoids, LMTK3 was significantly upregulated compared to normal counterparts. Treatment with C28 (an LMTK3 inhibitor) suppressed organoid cell viability, functionally validating LMTK3/FADS2 pathway dependency in breast cancer progression (33). In another study (34), organoid models are additionally employed to validate mechanisms originally defined at the molecular virology level. Because organoids provide a controlled and defined cellular environment, such perturbations enable direct assessment of causal relationships between molecular changes and biological responses. In this context, mechanistic interrogation and target discovery are closely coupled, as candidate molecular targets must be causally linked to disease-relevant phenotypes to achieve translational relevance.

Target identification can also be guided by literature-driven strategies. Prior understanding of disease-associated signalling pathways, receptor-ligand interactions, or conserved molecular mechanisms can inform rational target selection, particularly in settings where omics data or species-specific reagents are limited. Additionally, organoid-based mechanistic insights may also inform reinterpretation of clinical observations rather than only predict treatment outcomes.

From a translational perspective, organoid-based findings should be interpreted with realistic expectations. Many diseases are not driven by single causal genes but instead arise from dysregulated molecular and cellular networks, where disease phenotypes reflect shifts in network states rather than linear cause-effect relationships *in vivo* (35). However, organoid models do not recapitulate the full complexity of *in vivo* disease; rather, they define context-dependent boundary conditions under which specific pathways are sufficient or insufficient to drive disease-relevant phenotypes. For example, using the controlled keratinocyte-exclusive organoid model without secondary infection, the induction of Th2 cytokines enabled investigation of Th2 cytokine signaling within keratinocytes, helping to elucidate how this pathway contributes to cell-specific pathogenesis of atopic dermatitis pathogenesis (9). This context highlights the value of organoid systems as well-controlled, intermediate platforms, from simple to complex *in vitro* models, that retain key structural and functional similarities to native tissue. By offering these advantages, organoids serve as an indispensable tool for prioritizing causal mechanisms, bridging the critical gap between traditional 2D cell culture and animal studies.

## 5 Discussion and outlook

The rapid expansion of organoid technology across veterinary species and organ systems represents a major methodological advance for *in vitro* disease modelling. However, as discussed in this Perspective, current progress remains largely driven by model establishment rather than by hypothesis-driven investigation of disease mechanisms. As a result, many veterinary organoid studies remain descriptive and therefore limit their translational impact.

Nevertheless, organoid disease modelling has already demonstrated clear advantages beyond descriptive *in vitro* systems. Intestinal organoids derived from pigs and dogs have substantially accelerated investigations into enteric viral and bacterial infections by providing a controlled epithelial platform that allows rapid interrogation of pathogen entry (36, 37), barrier dysfunction (30, 38), and immune responses (31, 39), thereby reducing reliance on time-consuming and variable *in vivo* challenge models. In veterinary oncology, patient-derived tumour organoids have complemented conventional biomarker-driven approaches by enabling functional validation of pathway dependency and drug responsiveness. For example, canine bladder cancer organoids have been used to identify and functionally validate MEK pathway dependency, supporting the therapeutic evaluation of MEK inhibition in a disease-relevant context (29). Beyond mechanistic investigation, organoid systems have also enabled direct hypothesis testing by isolating epithelial-intrinsic responses to defined stimuli, such as cytokines (9, 40), toxins (22), or genetic manipulations (32).

**Figure 1:**
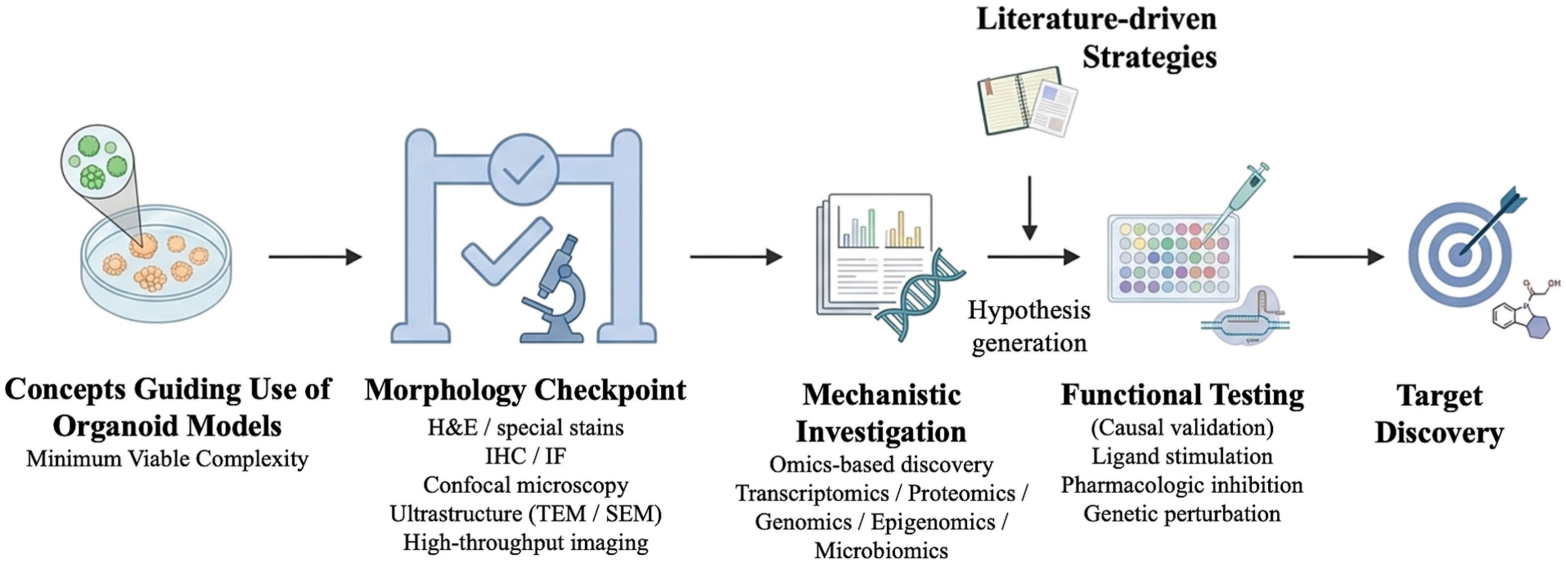
Framework workflow from pathogenesis to treatment through organoid disease modelling (Created in BioRender. Chen et al. (9) https://BioRender.com/0p3y8rj).

Within appropriately selected and validated models, organoid systems offer a valuable platform for disease-relevant target discovery and translational exploration (Figure 1). With the FDA’s 2025 recognition of validated organoid models for preclinical assessment to reduce animal testing (41), organoid disease models are expected to be increasingly integrated into the drug development process. In the framework outlined by Kang et al. (42), the use of model-omics data to guide organoid selection and the development of context-of-use assays by quantifying their ability to recapitulate clinically observed responses was highlighted. At the current stage of veterinary organoid research, conducting a sufficient number of proof-of-concept studies using components with well-characterized mechanisms of action is therefore encouraged to validate organoid disease model systems. Oclacitinib, a JAK1-preferential inhibitor, is approved for the clinical treatment of canine atopic dermatitis to control pruritus and inflammation (9), therefore represents an appropriate proof-of-concept compound for assessing whether Th2 cytokine-treated canine epidermal organoids capture pharmacologically relevant JAK–STAT pathway responses. Such studies can demonstrate the applicability of validated organoid models for pharmacological modulation and, potentially, large-scale drug screening, including pathway-specific ligand stimulation or inhibition using well-defined compounds.

Organoids facilitate knowledge exchange between human and veterinary medicine, and stimulate research and strengthen overall public health resilience. Reverse translational science was proposed and refers to a bidirectional process whereby clinical insights from patients guide early investigations in appropriate animal models of spontaneous disease, with the resulting data subsequently used to predict therapeutic efficacy and safety (43). As organoid technology has advanced more rapidly in human biomedical research, leveraging these innovations for a human-to-veterinary bidirectional translation provides unique opportunities to enhance veterinary disease modelling and therapeutic development. In addition, broader collaborative networks are also emerging to consolidate these efforts. For example, the Monash Biomedicine Discovery Institute (BDI) Organoid Program and the FHTTA Organoid Nexus in Australia provide valuable platforms that disseminate both foundational and emerging organoid technologies, thereby supporting and guiding veterinary researchers. European initiatives such as VetBioNet and ISIDORe similarly aim to strengthen collaboration, infrastructure, and resource sharing in organoid applications in veterinary and infectious disease research.

Medical research envisions the great potential of personalized treatments, with the aim of optimizing diagnoses and tailoring therapeutic strategies for individual patients (44). The preservation of inter-individual biological variability in primary cell-derived organoids represents a biological strength, particularly in veterinary medicine where disease heterogeneity is common and therapeutic responses are often unpredictable, such as in cancers and atopic dermatitis. Our preliminary studies investigating canine atopic dermatitis indicate that the Th2 signalling axis reveals inter-individual differences in pathway activation and the expression of key proteins among Th2 cytokine-treated epidermal organoids derived from healthy dog skin, suggesting that mechanisms predisposing to atopic disease may vary between animals. Building on this concept, canine primary epidermal organoids derived from atopic skin and exposed to Th2 cytokines may provide a platform for personalised drug screening by evaluating inter-individual therapeutic responsiveness.

Beyond drug discovery as a therapeutic strategy, organoid technology also holds potential applications in veterinary regenerative medicine and offers alternative solutions to intractable clinical problems. The use of organoid-derived cells has been explored in a canine disease model, a COMMD1-deficient dog model of inherited metabolic liver disease, where transplanted autologous liver organoids showed long-term survival (45). However, the broader application of regenerative medicine in veterinary practice remains constrained by several factors, including cost, ethical considerations, regulatory challenges, and uncertainties regarding long-term safety and efficacy. Nevertheless, large lab animal *in vitro* models such as dogs and pigs are expected to play an increasingly important role in translational regenerative medicine research (46).

Looking forward, organoid disease modelling presents emerging opportunities for more advanced therapeutic strategies in veterinary medicine by linking mechanistic insight, translational testing, and individualized therapeutic strategies with the guidance of clear biological questions.

## Data Availability

The original contributions presented in the study are included in the article/supplementary material, further inquiries can be directed to the corresponding author.
